# Probiotic edible films as a new strategy for developing functional bakery products: The case of pan bread^☆^

**DOI:** 10.1016/j.foodhyd.2014.01.023

**Published:** 2014-08

**Authors:** Christos Soukoulis, Lina Yonekura, Heng-Hui Gan, Solmaz Behboudi-Jobbehdar, Christopher Parmenter, Ian Fisk

**Affiliations:** a Division of Food Sciences, School of Biosciences, University of Nottingham, Sutton Bonington Campus, Loughborough, Leicestershire LE12 5RD, United Kingdom; b Nottingham Nanotechnology and Nanoscience Centre, University of Nottingham, University Park, Nottingham NG7 2RD, United Kingdom

**Keywords:** Probiotic, Viability, Functional foods, Bread, In-vitro digestion, Novel processing

## Abstract

In the present paper, a novel approach for the development of probiotic baked cereal products is presented. Probiotic pan bread constructed by the application of film forming solutions based either on individual hydrogels e.g. 1% w/w sodium alginate (ALG) or binary blends of 0.5% w/w sodium alginate and 2% whey protein concentrate (ALG/WPC) containing *Lactobacillus rhamnosus* GG, followed by an air drying step at 60 °C for 10 min or 180 °C for min were produced. No visual differences between the bread crust surface of control and probiotic bread were observed. Microstructural analysis of bread crust revealed the formation of thicker films in the case of ALG/WPC. The presence of WPC improved significantly the viability of *L. rhamnosus* GG throughout air drying and room temperature storage. During storage there was a significant reduction in *L. rhamnosus* GG viability during the first 24 h, viable count losses were low during the subsequent 2–3 days of storage and growth was observed upon the last days of storage (day 4–7). The use of film forming solutions based exclusive on sodium alginate improved the viability of *L. rhamnosus* GG under simulated gastro-intestinal conditions, and there was no impact of the bread crust matrix on inactivation rates. The presence of the probiotic edible films did not modify cause major shifts in the mechanistic pathway of bread staling – as shown by physicochemical, thermal, texture and headspace analysis. Based on our calculations, an individual 30–40 g bread slice can deliver approx. 7.57–8.98 and 6.55–6.91 log cfu/portion before and after in-vitro digestion, meeting the WHO recommended required viable cell counts for probiotic bacteria to be delivered to the human host.

## Highlights

• A new strategy for the inclusion of probiotics in baked cereal products is presented.
• Surface coated edible films effectively protected *Lactobacillus rhamnosus* GG on pan bread.
• Whey proteins reduced *L. rhamnosus* GG loss during drying and storage.
• Sodium alginate based films offered the best protection during in-vitro digestion.

## Graphical abstract

**Figure:**
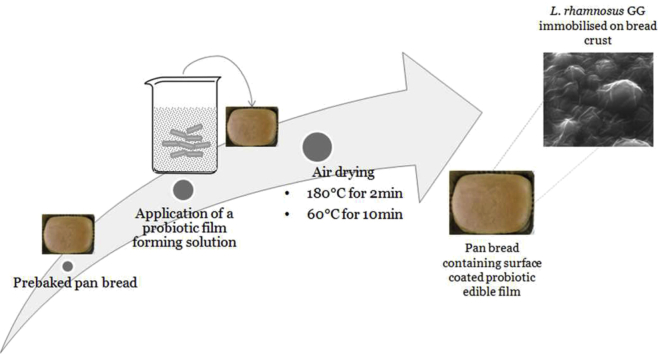

## 1 Introduction

Probiotics are defined as live organisms that when administered in adequate amounts (>6–7 log cfu/g) confer health benefits to the host (FAO/WHO 2002). Although the functionality of the probiotics is generally strain dependent, health benefits including regulation of the gastrointestinal tract, stimulation of the immune system, reduction of serum cholesterol levels and lactose intolerance and prevention of cancer and cardiovascular disease have been reported (Saad, Delattre, Urdaci, Schmitter, & Bressollier, 2013). The growth of the probiotic food segment has been remarkable over the last decade with dairy products i.e. yogurt, ice cream, cheese and milk, juices and beverages and infant formulations being predominant (Euromonitor, 2012). Processing conditions during production can lead to significant losses of probiotic viability due to heat, mechanical or osmotic stress induced cellular injuries (Bustos & Bórquez, 2013; Fu & Chen, 2011). Several strategies have been extensively researched over the last years to overcome these processing obstacles to establish maximum viability of probiotics throughout the entire production cycle including product storage, market distribution and also during consumption i.e. under gastric juice and intestine bile salt conditions. To date, microencapsulation of probiotics in spray or freeze dried matrices and the inclusion of probiotics in biopolymer based microcapsules is the most common route for production of viable probiotic bacteria in real food systems (Burgain, Gaiani, Linder, & Scher, 2011; Dianawati, Mishra, & Shah, 2013; Fritzen-Freire et al., 2012; Malmo, Storia, & Mauriello, 2013; Soukoulis, Behboudi-Jobbehdar, Yonekura, Parmenter, & Fisk, 2013; Yonekura, Sun, Soukoulis, & Fisk, 2013).

The term edible films refers to thin layered structures of biopolymer composition that can be consumed and are usually applied onto the surface of food products by dipping, spraying or brushing (Bourtoom, 2008). The application of edible films in food has previously been shown to be effective for the control of shelf-life by slowing detrimental reactions e.g. enzymatic, physical and chemical by raising a thermodynamical or physical barrier that retards water vapour, oxygen and solutes mobility (Falguera, Quintero, Jiménez, Muñoz, & Ibarz, 2011). Over the last decade significant research has been carried out into the production of edible films with good barrier and mechanical properties and high levels of biodegradability. Recently, edible films and coatings have been introduced as efficient carriers for the delivery of several bioactive compounds e.g. vitamins, antioxidants and probiotics in food systems (Kanmani & Lim, 2013; López de Lacey, López-Caballero, Gómez-Estaca, Gómez-Guillén, & Montero, 2012). Furthermore, the application of edible films as growth inhibitors of microbial spoilage and pathogens has been also reported. Regarding probiotic edible films, López de Lacey et al. (2012) recently showed that the immobilisation of *Bifidobacteria bifidum* and *Lactobacillus acidophilus* in gelatine based matrices could provide a good protection against viability loss for at least six days of storage under chilled conditions e.g. 2 °C and Kanmani and Lim (2013) reported that the viability of multiple probiotic strains e.g. *Lactobacillus reuteri* ATCC 55730, *Lactobacillus plantarum* GG ATCC 53103 and *L. acidophilus* DSM 20079 in starch–pullulan based edible films was strongly influenced by the pullulan to starch ratio and storage temperature.

Bakery products including breads are staple foods comprised of several major components (complex carbohydrates, insoluble dietary fibre, proteins, lipids, minerals and vitamins) in varying proportions and with varying physical interactions and structures. Increasing awareness by consumers of healthier bakery products has led to a significant improvement in the health aspects of bread e.g. mineral and vitamin fortified breads, salt, fat and sugar reduced formulations and gluten or allergen free products (Belz, Ryan, & Arendt, 2011; Bigliardi & Galati, 2013; Gallagher, Gormley, & Arendt, 2004). Recent advances in bread making include seeking new ways of delivering heat sensitive bioactive materials, one such example being that of probiotics. Altamirano-Fortoul, Moreno-Terrazas, Quezada-Gallo, and Rosell (2012) described a strategy for delivering probiotics in bread using edible coatings and anhydrobiotics. More specifically, successive layers of starch based coatings and microencapsulated probiotic bacteria have been applied on the surface of part-baked breads followed by a short baking process. In their approach, Altamirano-Fortoul, Le-Bail, et al. (2012) and Altamirano-Fortoul, Moreno-Terrazas, et al. (2012) reported that bread loaves retained relatively high amounts of viable bacteria after the baking process (2.4–3.05 × 10^7^ log cfu/g) and a loss of approx. 1.0–1.4 log cfu/g was observed after 24 h of storage at room temperature.

Based on our recent studies (Soukoulis and Fisk, unpublished data) single probiotic strain edible films comprised of selected biopolymers including proteins, polysaccharides and prebiotics provide a good substrate for the retention of *Lactobacillus rhamnosus* GG, for at least 10 days, under room temperature storage conditions. Edible films therefore seem a very promising route for the delivery of probiotics in food systems where probiotics cannot be incorporated following the conventional approach of direct inoculation. In the present work we demonstrate for the first time the application of probiotic edible films on a staple bakery system i.e. pan bread. Two edible films were applied on the crust of pre-baked bread and formed using rapid convective drying (60 °C and 180 °C). The structural, mechanical and microbiological characteristics of the bread crust were fully investigated together the performance of the bread systems under simulated gastro-intestinal conditions.

## 2 Materials and methods

### 2.1 Materials

In the present work a probiotic strain with well-established probiotic functionality, namely *L. rhamnosus* GG strain (E-96666VTT, Espoo, Finland), was used. Sodium alginate (RF 6650, Protanal^®^, Drammen, Norway) and whey protein concentrate (Lacprodan^®^ DI-8090, Visby, Denmark) were kindly provided as a gift by FMC Biopolymers Ltd. and Arla A/S respectively. Glycerol (purity > 99%) was used as plasticising agent (Sigma Aldrich, Gillingham, UK).

### 2.2 Stock culture preparation and growth conditions of *L. rhamnosus* GG

One milliliter of sterile phosphate buffer saline pH 7.0 (Dulbecco A PBS, Oxoid Ltd., Basingstoke, UK) was added to the lyophilised culture of *L. rhamnosus* GG and after adequate mixing the bacterial aliquot was transferred and streaked on set MRS-agar medium (MRS Agar, Oxoid Ltd., Basingstoke, UK). The petri dishes were left to grow under anaerobic conditions in hermetically sealed plastic containers with Anaerogen^®^ (Oxoid Ltd., Basingstoke, UK) at 37 °C for 48 h. A small amount of the colonies were collected with a sterilised loop and suspended in the cryo-medium of Microbank systems (Pro-Lab Diagnostics UK, Merseyside, UK), the plastic bead cultures were stored in a freezer at −80 °C (Ananta, Volkert, & Knorr, 2005).

Five beads of the deep frozen cultures were placed in 500 mL of MRS broth (Oxoid Ltd., Basingstoke, UK) and incubated for 48 h at 37 °C under anaerobic conditions in plastic jars containing AnaeroGen^®^ (Oxoid Ltd., Basingstoke, UK). The final broth was transferred under aseptic conditions into 50 mL sterile centrifuge tubes (Sarstedt Ltd., Leicester, UK) and centrifuged at 3000 g for 5 min. After centrifugation, the supernatant was discarded and the harvested cells in the form of pellets were washed twice using phosphate buffer saline pH 7.0.

### 2.3 Preparation of the film forming solutions

Two film forming solutions were prepared by dispersing the dry materials (1% w/w sodium alginate or a blend comprising of 0.5% w/w sodium alginate and 2.0% w/w whey protein concentrate) in distilled water at 25 °C under agitation for 1 h to allow full hydration. After the addition of the plasticiser at the level of 50% of polysaccharides total solids, the biopolymer-glycerol dispersions were heated to 80 °C for 30 min to allow full dissolution and hydration and to destroy any residual microflora. Then, the film forming solutions were cooled down to 25 °C and isothermally stored until inoculation with *L. rhamnosus* GG pellets. Six pellets (corresponding to 300 mL of the broth culture) were suspended in 80 mL of the film forming solutions and degassed using a vacuum pump for 5 min.

### 2.4 Bread preparation

The bread dough was prepared following the procedure as described by Giannou and Tzia (2007) having the following composition (on % w/w of flour basis): strong wheat flour (100), water (60), lyophilised baker's yeast (2), crystalline sucrose (4), salt (2), sunflower oil (3) and ascorbic acid (0.01). The ingredients were mixed for 10 min at the lowest speed using a lab scale mixer. The dough was bulk proved for 80 min in a proving chamber (Salva 22 Tray Cabinet Prover, Lezo, Spain) at 40 °C, 75% RH, divided into individual samples of 150 g, shaped, placed in aluminium pans and proved under the same conditions for 30 min. Then, the samples were baked in a preheated oven at 180 °C for 35 min implementing a steaming step for the first 7 min of baking. The bread loaves were taken off the pans, placed on a metallic rack to cool for 30 min. Oven used throughout was Tom Chandley 12 tray high crown deck oven, MK6 controls, Manchester, UK.

A small amount of probiotic edible film forming solution was applied and uniformly distributed by brushing on the crust of the bread loaves. Bread samples were weighed before and after the film forming solutions application to calculate the exact amount (approx. 1.00 ± 0.07 g) and the initial total viable count of *L. rhamnosus GG* on bread crust. The probiotic breads were then rapidly dried following two different regimes: a) a low temperature drying at 60 °C for 10 min in an air circulating drying chamber, and b) a high temperature-short time drying viz. 180 °C for 2 min using the baking oven without the steaming step.

After the completion of the drying step, the bread samples were left to cool to room temperature (25 ± 1 °C) and then were packaged in the thermo-sealed polyethylene bags. All bread samples were stored in temperature controlled chambers at 25 °C and 55% RH. Physicochemical, textural and microbiological analyses were then run 2 h, 24 h, 48 h, 96 h, 144 h and 168 h after the end of the baking process, whilst in-vitro digestion was run on freshly baked loaves (2 h).

### 2.5 Moisture and water activity

The residual water content (g/100 g) was calculated according to AACC method 44-15.02. Small amounts of pre-weighed bread crust or crumb (approx. 1.5 g) were transferred to aluminium pans and dried at 105 °C for 24 h to reach weight equilibrium. Residual water content was calculated according to formula (1).

(1) $$\%\;\text{residual}\;\text{water}\;\text{content}=100\times \frac{w_{i}-w_{f}}{w_{i}}$$

Where *w*_*i*_, and *w*_*f*_ are the weights of the dry probiotic formulations prior and after dehydration at 105 °C.

Water activity of the bread crust and crumb was determined using an AquaLab water activity meter (AquaLab, 3TE, Decagon, USA).

### 2.6 Colour characteristics

The colour of the bread crust samples was determined using a Hunterlab (Reston, USA) colorimeter. The CIELab color scale was used to measure the L* (black to white), a* (red to green), and b* (yellow to blue) parameters. The total colour difference ΔE* between the control sample (uncoated bread crust) and each individual probiotic edible film was calculated according to the formula (2):

(2) $$\Delta E^{*}=\sqrt{{\left(\Delta L^{*}\right)}^{2}+{\left(\Delta a^{*}\right)}^{2}+{\left(\Delta b^{*}\right)}^{2}}$$

where ΔL*, Δa*, Δb*, are the brightness, redness and yellowness intensity difference from the control sample.

### 2.7 Bread crust textural analysis

The determination of textural characteristics was carried out using a Texture Analyser (TA-XT exponent, Stable Microsystems, Ltd., Godalming, UK) using the method described by Altamirano-Fortoul and Rosell (2011) modified as proposed by Altamirano-Fortoul, Hernando, and Rosell (2013) in the case of thin crust bread systems. A cylindrical probe of 2 mm (P/2) was used whereas the measuring conditions were: 10 mm/s pre-test speed, 10 mm/s cross speed and 10% of crust compression (strain). From the force–deformation curves of three individual bread loaves the following characteristics were calculated: Failure force (*N*) = the peak force during crust puncture test; failure deformation = the probe travelling distance (mm) from the crust puncture startpoint (*d*); crust rupture work (N*s) = the area (*A*) under the force–deformation curve; average puncturing force (*N*) = the ratio of area to failure deformation (*A*/*d*).

### 2.8 DSC measurements

Samples of bread crust (2 h and 7 d after baking) and adjacent to crust bread crumb (2 h after baking) were pressed, weighed in a hermetically sealed aluminium pan and analysed using a Perkin-Elmer DSC. Calibration of the DSC was achieved using pure indium. Samples were rapidly cooled (10 °C/min) from 25 to −80 °C, held isothermally for 2 min and heated from −80 to 180 °C at the rate of 5 °C/min. A second cooling–heating step (from −80 to 180 °C) was repeated following the previous cooling/heating rates in order to determinate the glass transition temperature of the analysed bread crust and crumb samples. From the obtained thermographs for the bread crumb samples the glass transition temperature, ice fusion enthalpy, onset and midpoint of ice fusion, were calculated. In the case of the bread crust samples, the glass transition temperature and the amylopectin retrogradation enthalpy were also calculated as function of storage time (Ribotta & LeBail, 2007).

### 2.9 Morphological characterisation of bread crust

Bread crust samples were cut in circular shapes (⍉10 mm) and carefully deposited onto carbon tabs (Agar Scientific, Stansted, UK), coated with carbon (Agar turbo carbon coater) and placed on the stage of a FEI Quanta 3D 200 dual beam Focused Ion Beam Scanning Electron Microscope (FIM-SEM, FEI, Hillsboro, USA). Images were acquired using secondary electron imaging at an accelerating voltage of 5–15 kV.

### 2.10 APCI-MS and SPME-GC-MS headspace analysis of bread crust during storage

A MS Nose interface (Micromass, Manchester, UK) fitted to a Quattro Ultima mass spectrometer (Milford, Waters, UK) was used for the static headspace analysis of bread samples. 50 g samples were placed in 40 mL vials fitted with a one port lid. After a 30 min equilibration period at room temperature (20 °C), the headspace was drawn into the APCI-MS source at a rate of 5 mL/min. The samples were analysed in full scan mode, monitoring ions of mass to charge (*m*/*z*) ratios from 20 to 200. The intensity of these ions was measured at cone voltage of 50 V, source temperature of 75 °C and dwell time of 0.5 s. All analyses were run in triplicate and were based on the methods of Gan, Soukoulis, and Fisk (2014).

### 2.11 Enumeration of the bacteria

One milliliter of the probiotic film forming solutions was suspended in sterile PBS and vortexed for 30 s to ensure adequate mixing. For the recovery of *L. rhamnosus* GG from the bread crust sample the method described by López de Lacey et al. (2012) with minor modifications was adopted. More specifically, 1 g of *L. rhamnosus* GG containing bread crust samples were transferred to 9 mL of sterile PBS and left to hydrate and dissolve under constant agitation in an orbital incubator at 37 °C for 1 h. In both cases, the resulting solutions were subjected to serial dilutions using phosphate buffer saline. Each dilution was pour plated on a MRS agar (MRS Agar, Oxoid Ltd., Basingstoke, UK) and the plates were stored at 37 °C for 72 h under anaerobic conditions to allow colonies to grow. Enumeration of the bacteria was performed in triplicate following the standard plating methodology (Champagne, Ross, Saarela, Hansen, & Charalampopoulos, 2011) and the total counts of the viable bacteria were expressed as log colony forming units per gram (log CFU/g).

The survival rate of the bacteria throughout the film forming solution drying process was calculated according to the following equation (3).

(3) $$\%\;\text{viability}=100\times \frac{N}{N_{0}}$$

Where: *N*_0_, *N* represent the number of viable bacteria prior and after the implemented drying process (Ananta et al., 2005).

### 2.12 In-vitro digestion

Probiotic bread crust systems and free bacteria with or without bread crust were compared for their ability to survive in-vitro digestion simulating the human gastric and intestinal environments. The method was based on a previously published procedure (Yonekura & Nagao, 2009), with modifications to better resemble the gastric pepsin activity (1600 unit/mL) and duodenal bile salt concentration (4.4 g/L) found in human aspirates (Faye, Tamburello, Vegarud, & Skeie, 2012; Reppas & Vertzoni, 2012). Three-hundred milligrams of bread crust or the equivalent counts of viable bacteria (pelleted from MRS broth and washed once with PBS) were suspended in 2.5 mL saline in 30 mL glass vials, then 2.5 mL of double-concentrated simulated gastric juice was added (pH 2.5, 3200 units/mL pepsin, 7.2 mmol/L CaCl_2_, 3 mmol/L MgCl_2_, 98 mmol/L NaCl, 24 mmol/L KCl, and 12.8 mmol/L KH_2_PO_4_). The vials were screw-capped under a stream of N_2_ and incubated for 1 h at 37 °C and constant magnetic stirring at 130 rpm/min (gastric digestion). Pancreatin and bile salts were dissolved in 0.1 mol/L NaHCO3, added to the gastric digesta (final concentrations were 4.4 g/L bile salts and 4 mg bile salts), and incubated for 2 h (Yonekura et al., 2013). Aliquots of the digesta (1 mL) were diluted in 9 mL of PBS and plated on MRS agar for enumeration of viable bacteria, as described above.

### 2.13 Statistical analyses

Two-way ANOVA followed by Duncan's post hoc means comparison (*p* < 0.05) test was performed to evaluate the main effects of edible film system and drying conditions on the physicochemical, mechanical and microbiological data. Repeated measurements ANOVA was applied for the investigation of storage time effect on viable counts of *L. rhamnosus* GG. All statistical calculations were performed using the MINITAB release 16 statistical software (Minitab Inc., PA, USA).

## 3 Results and discussion

### 3.1 Appearance and morphological characterisation of the bread crust

**Fig. 1:**
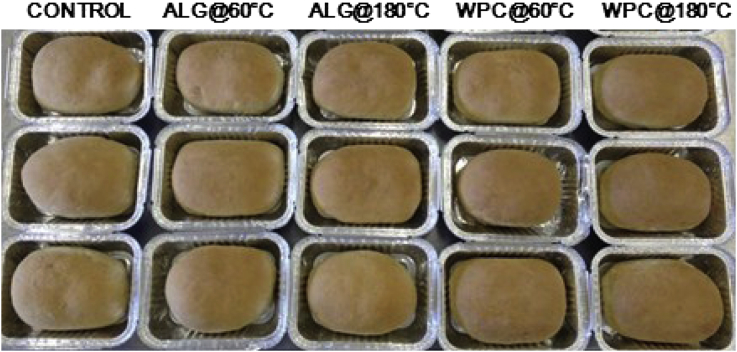
Appearance of the crust of conventional and probiotic bread loaves (coated with sodium alginate (ALG) or sodium alginate–whey protein concentrate (ALG/WPC) edible films and air dried either at 60 °C for 10 min or 180 °C for 2 min) using the probiotic edible films strategy.

In Table 1 are displayed the colour characteristics of the probiotic bread crusts. The presence of the edible films impacted significantly (*p* < 0.001) most of the colour characteristics of the bread crusts (L*, a* and ΔΕ*), with the control being characterised as having the highest luminosity and lowest red colour component of the hue, as reflected by a* values. No significant differences were observed between the control and the samples containing the alginate based films whilst the bread loaves treated with WPC based films and dried at oven temperature were characterised by the highest yellow component of the hue intensity (b*, *p* < 0.05). In addition, the presence of whey protein in the film was accompanied by darker and higher red colour intensity particularly in the case of the most aggressive drying conditions (180 °C for 2 min). The former could be attributed to the formation of colour products e.g. melanoidins via Maillard reaction chemistries occurring between the whey protein and lactose – this is generally favoured at high pH conditions (pH 6–7) as recently reported (Wang, Bao, & Chen, 2013). The latter might be of particular importance not only because of changes on the visibly perceived colour of the finished products (ΔΕ*) but also because of the potential formation of products with free radical scavenging activities that could potentially protect entrapped bacteria from free radical-driven oxidative damage. Only the bread crust treated with WPC and dried at oven temperature had a ΔΕ* value higher than 3, ΔΕ* values of greater than 3 have been reported as a visual threshold for the distinction of colour changes by the human eye (Martínez-Cervera, Salvador, Muguerza, Moulay, & Fiszman, 2011). Images of the bread surface are shown in Fig. 1 and show that the application of the probiotic edible films was primarily associated with higher brown colour compared to the control breads. The latter has been reported as being an important quality trait which is typically related with production practices such as steaming during baking, that can promote starch gelatinisation and dextrin caramelisation (Altamirano-Fortoul, Le-Bail, Chevallier, & Rosell, 2012). However, the visual differences between film type and drying conditions are only distinguishable under close visual inspection.

**Fig. 2:**
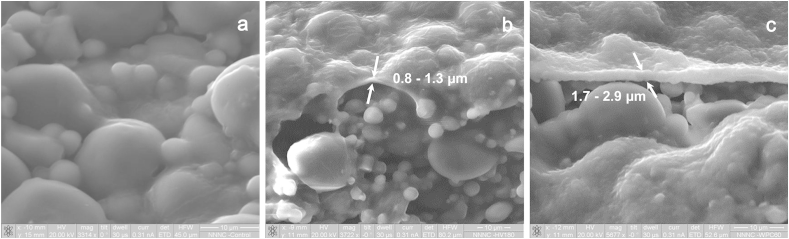
SEM analysis of the crust of conventional (a) and probiotic pan bread loaves dried at 60 °C after film application (b,c). Probiotic breads were coated with either sodium alginate (b) or sodium alginate–whey protein concentrate probiotic edible films. Scale bar = 10 μm.

**Fig. 3:**
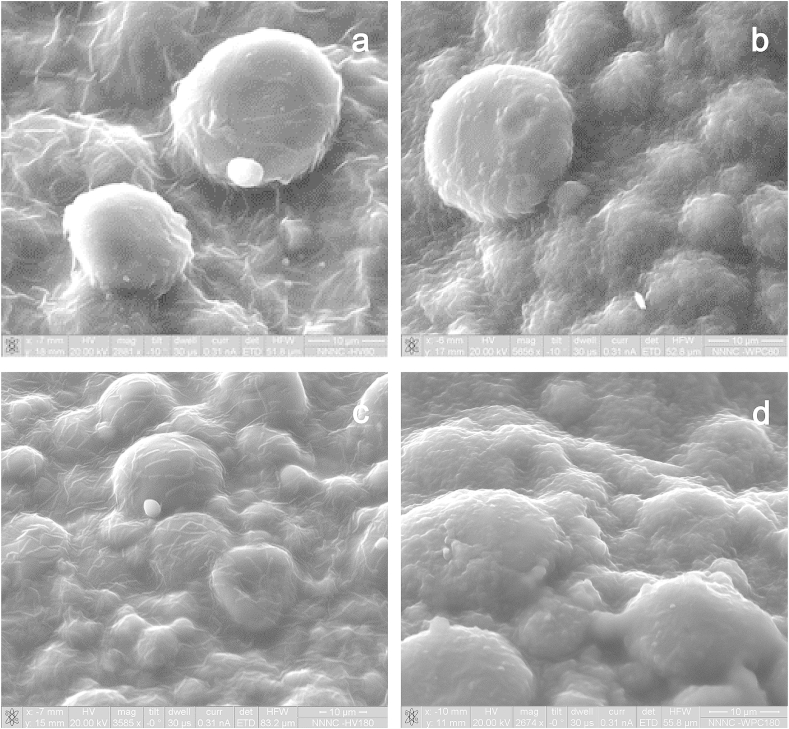
SEM analysis of probiotic pan bread crust samples coated with sodium alginate edible films dried at 60 °C (a) or 180 °C (c) or sodium alginate–whey protein concentrate edible films dried at 60 °C (b) or 180 °C (d). Scale bar = 10 μm.

Scanning electron microscopy of the upper surface of the bread allowed the evaluation of the colloidal differences on the crust surface samples between the control and edible films coated samples (Fig. 2), and the influence of composition and drying conditions (Fig. 3). As was expected, the application of the edible films did not modify the main structural aspects of the bread crust which contained partially gelatinised starch granules and a gluten formed structural network (Altamirano-Fortoul, Moreno-Terrazas, et al., 2012). The addition of the edible films resulted in the formation of an independent biopolymer layer over the surface of the bread; the thickness was dependent on the amount and type of solid used in the film forming solution. Sodium alginate probiotic films had a significantly lower thickness (0.8–1.3 μm) compared to the WPC containing films (1.7–2.9 μm). The low thickness of the sodium alginate films made possible to visualise the probiotic bacteria embedded in the dried polysaccharides matrix (Fig. 3a,c). On the contrary, the use of the WPC-sodium alginate films (Fig. 3b,d) was associated with the formation of a thicker layer comprised of protein aggregates proposed to be formed during the heat induced denaturation of the whey proteins (Hussain, Gaiani, Jeandel, Ghanbaja, & Scher, 2012). It is very interesting that in the latter case it was not possible to view the bacterial rods implying that the WPC based probiotic films might provide a better surface coverage of the bacteria and potentially enhance their resistance against the toxic extrinsic conditions e.g. oxygen and water vapour. Regarding the impact of the drying method, SEM micrographs revealed no significant differences between the coated bread crust samples dried at 60 or 180 °C.

### 3.2 Physical properties of bread crust and crumb during storage

**Fig. 4:**
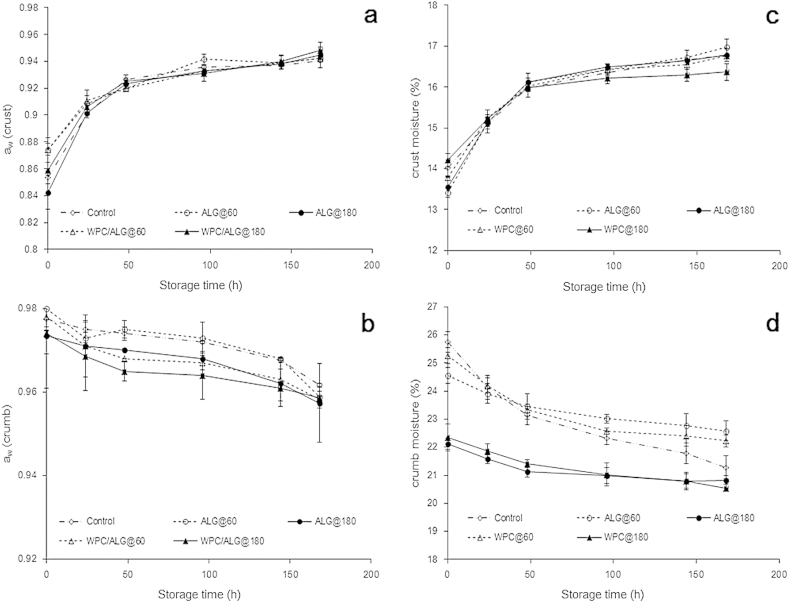
Changes in water activity (a,b) and residual water content (c,d) of pan bread crust coated with sodium alginate (ALG) or sodium alginate–whey protein concentrate (ALG/WPC) edible films and air dried either at 60 °C for 10 min or 180 °C for 2 min, and crumb samples during 7 days (168 h) of storage at room temperature.

Water vapour mass transfer between the crust and crumb interface is the driving force of moisture distribution from the interior to the exterior of bread loaves (Gray & Bemiller, 2003). Residual water content and water activity of probiotic bread samples were tested 2 h after baking and over seven days storage. Expectedly, moisture and water activity of bread crust samples varied significantly (*p* < 0.001) during storage (Fig. 4). Water activity of bread crust samples increased from 0.85 to 0.95 over 7 days of storage and there was a minor change of bread crumb water activity over the storage period from 0.97 to 0.96. There was no significant impact of edible film type or drying method on water activity and moisture content of bread crust samples. On the contrary, the drying method affected significantly (*p* < 0.05) the water activity and residual water content of bread crumb, with the oven temperature drying step inducing the highest reduction in water content. However, similarly to the bread crust samples, the composition of the film did not result in any major changes to the bread crumb samples. Our results are generally in accordance with the findings of Altamirano-Fortoul, Le-Bail, et al. (2012) and Altamirano-Fortul, Moreno-Terrazas, et al. (2012) who reported that the application of an edible coating containing spray dried probiotic microcapsules did not modify significantly the moisture transport phenomena in probiotic thick crust bread.

### 3.3 *L. rhamnosus* GG viability during storage

**Fig. 5:**
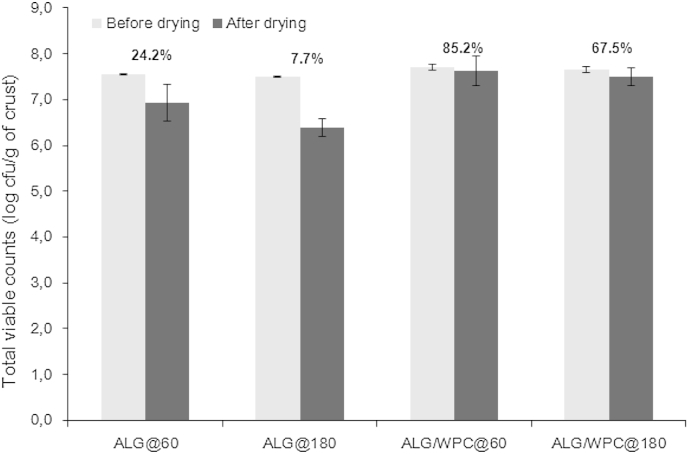
Viability of *L. rhamnosus* GG (log cfu/g of bread crust) in pan bread crust samples coated with sodium alginate (ALG) or sodium alginate–whey protein concentrate (ALG/WPC) films before and after air drying at 60 °C for 10 min or 180 °C for 2 min.

The probiotic bread samples were microbiologically tested from 2 h after baking to 7 days storage in order to evaluate the retention of probiotic viability within the systems whilst exposed to the extrinsic (water vapour, oxygen, heat induced injuries) and intrinsic (osmotic stress and physical state changes due to bread staling) stresses that occur in typical storage conditions. As it can be seen in Fig. 5, the viability of the bacteria throughout the film drying step was significantly influenced only by the composition of the film forming solutions (*p* < 0.05). More specifically, a lower viability percentage was determined in the case of the breads coated with sodium alginate compared to those containing WPC (ANOVA mean values were 15.9% and 76.3% respectively). It is well established that convective drying of probiotics induces a significant loss in viability due to changes in their cellular structure i.e. phase transitions of the lipid bilayer of the membranes can lead to membrane rupture and leakage of cytoplasmic material (Fu & Chen, 2011). Moreover, the type of the material used for the encapsulation or immobilisation of the bacterial cells might also cause significant injuries due to osmotic stress. For example, polysaccharides such as pectin, cellulosics or alginates have been reported to impact the viability of bacteria cells both throughout the drying process and storage period (Bustos & Bórquez, 2013; Cook, Tzortzis, Charalampopoulos, & Khutoryanskiy, 2012; Yonekura et al., 2013). Furthermore, we have recently shown that changing the type of protein within a drying medium can impact the thermal and osmotic stresses through the ability of certain proteins to inhibit deteriorative chemical or enzymatic reactions e.g. lipid oxidation of membranes and hinder lethal phase transition during the drying process (Soukoulis et al., 2013). In addition, Burgain et al. (2013) investigated the in-vitro interaction between probiotic bacteria and milk proteins (micellar casein, native or denaturated whey proteins) and observed that *Lactobacilli* including *L. rhamnosus* GG, exhibits the ability to interact through their adhesive features, consisting of exopolysaccharides (EPS) or proteins, with whey proteins via electrostatic, steric or short-range forces, thereby improving their viability rates in dairy based food matrices. Surprisingly, the drying method did not confer any significant effect on the viability of the bacteria (*p* = 0.14) indicating that the application of the edible films at the final stage of the baking process followed by a rapid cooling of the bread loaves could be promising for the production of probiotic bakery products based on the edible film technology.

**Fig. 6:**
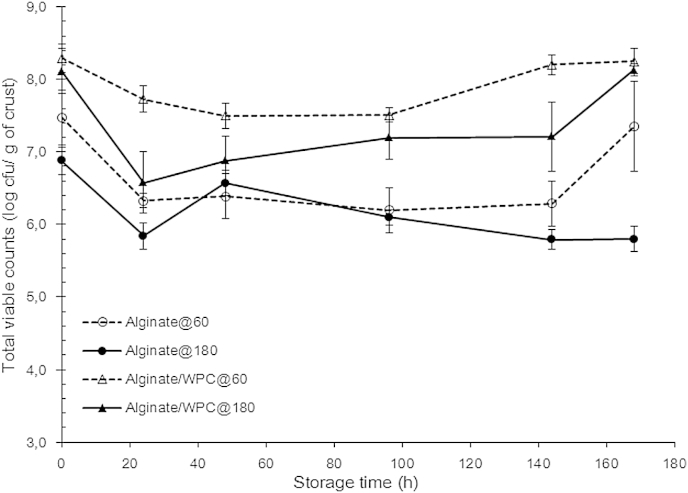
Viability of *L. rhamnosus* GG in pan bread crust stored for 7 days (168 h) at room temperature and coated with sodium alginate (ALG) or sodium alginate–whey protein concentrate (ALG/WPC) edible films and air dried either at 60 °C for 10 min or 180 °C for 2 min.

The fresh bread samples, after equilibrating for 2 h at room temperature, were placed in sealed polyethylene bags and stored for 7 days, the losses of *L. rhamnosus* GG through the entire storage period are shown in Fig. 6. The viability loss of three out of four bread systems (ALG@60, ALG/WPC@60 and ALG@180) exhibited a similar pattern as a function of storage time. More specifically, all three samples demonstrated a rather steep decrease in cell counts after 24 h of storage, followed by reduction of the inactivation rate over the successive 48 h reaching a latent-like state (plateau). Then, depending on the film forming material, a gradual recovery of the viability of *L. rhamnosus* GG was achieved followed by a clear growth stage during the last two days of storage (6th and 7th day). Only the system containing the *L. rhamnosus* GG immobilised in the sodium alginate matrix dried at oven temperature did not reveal any obvious sign of viability increase. Our results suggest that the direct inoculation and application of edible films with probiotics to bread crust can be considered an appropriate strategy for the production of functional bakery products. Although further studies are required in order to fully understand the interaction of probiotics with the individual components and the environmental conditions of the crust matrix; it appears that the composition of the edible films together with the physical changes that take place during storage i.e. bread staling and moisture migration from the core to the bread surface are both influential on probiotics viability.

The viability of *L. rhamnosus* GG in the bread crust appears to be strictly associated with the interactive relationship between the film forming material e.g. type of biopolymers used and the drying method implemented. Thus, it is implied that the implementation of mild drying methods combined with ingredients that favour the interaction with *L. rhamnosus* GG cells affect, not only the inactivation rates during the early storage stages, but also the ability of the strain to grow during latter storage. The latter is of particular importance not only because it modulates the shelf-life of the finished product (particularly in the case of modified atmosphere packaged products) but also because it significant impacts the potential of this technology as a viable route to probiotic inclusion in food materials. *L. rhamnosus* GG losses observed during the first day of storage were well correlated (*r* = −0.855, *p* < 0.001) with the amount of water migrated to the bread crust, and thus these losses could be mainly attributed to osmotic stress. The diminishing effect on the inactivation of *L. rhamnosus* GG observed during the successive six days seems to be associated with the hindrance of osmotic stresses and the establishment of acceptable conditions (*a*_*w*_ > 0.91) for the growth of *Lactobacilli* (FAO, 1998).

### 3.4 In-vitro digestion of the bread crust

**Fig. 7:**
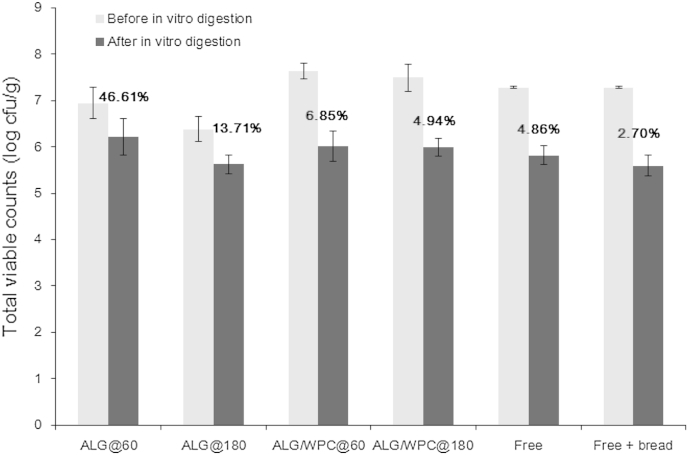
Viability of *L. rhamnosus* GG in bread crust samples after in vitro digestion (ALG = sodium alginate, WPC = whey protein concentrate, free = free cells of *L. rhamnosus* GG in phosphate buffer saline, free + bread = blend of free cells of *L. rhamnosus* GG and uncoated bread crust in phosphate buffer saline).

Bread crust samples coated with probiotic edible films were evaluated for their ability to stabilise probiotic cell viability under simulated gastrointestinal conditions (Fig. 7). Two systems of free bacteria (with or without added plain bread crust) harvested from MRS broth and washed with saline solution were subjected to in-vitro digestion in order to assess the impact of the edible film and bread crust matrix on the viability of probiotic cells. Our results showed that the presence of bread crust matrix does not provide any significant (*p* = 0.46) protection of the free probiotic cells against the simulated gastrointestinal conditions. More specifically, there was a reduction in viable cell counts from 1.47 to 1.69 log cfu/g for the free cell systems in the absence or presence of bread crust in the saline solution. The latter is very interesting as it implies that the viability of *L. rhamnosus* GG after the simulated digestion of the probiotic bread crust samples is predominantly affected by the presence of the film whilst the protective role of the bread matrix against the simulated gastrointestinal conditions is relatively minor (differences in the viabilities of *L. rhamnosus* GG in the free cell systems with or without bread crust were not statistically significant). The inclusion of *L. rhamnosus* GG in the sodium alginate films provided a significantly (*p* < 0.01) higher protection (the viable counts decrease ranged from 0.71 to 0.75 log cfu/g) throughout in-vitro digestion of probiotic cells compared to the systems coated with the WPC (1.49–1.61 log cfu/g) based edible films.

It is well known that the structure and disintegration rate of encapsulating systems under gastrointestinal conditions regulates their usefulness as carriers for the delivery of probiotic efficacy to the human host (Cook et al., 2012). In the present study, the pre-treatment of the bread crust systems with the saline solution would be expected to induce at least a partial disintegration of the applied edible films (both film types are easily soluble) thereby promoting the release of bacterial cells from the crust surface to the bulk aqueous phase. This suggests that the physicochemical and structural changes of the biopolymers (polysaccharides and whey proteins) due to the pH and ionic strength changes during in-vitro digestion impacted the viability of *L. rhamnosus* GG. Sodium alginate is well known for its ability to form strong gels in the presence of calcium ions in both neutral and acidic conditions through diffusion or internal setting (Helgerud, Gåserød, Fjæreide, Andersen, & Larsen, 2010). Due to the rather negligible amount of calcium ions in the saline-bread crust systems, the cross-linking of sodium alginate is not expected. The addition of gastric juice triggers the complexation of calcium ions (present in the pepsin solution) with sodium alginate and the formed gel encloses the bacterial cells found in the bulk aqueous phase. The reduction of viable bacteria counts in the case of the ALG@180 systems can be attributed to heat injuries induced during oven film drying and not with changes of the functionality of sodium alginate under the acidic conditions. Indeed, it has been reported that the severe convective drying processes such as high temperature air or spray drying can dramatically alter the stability of probiotic bacteria under subsequent gastrointestinal and osmotic stress conditions (Fu & Chen, 2011). On the contrary, the presence of whey proteins in the WPC based bread crust samples seems to hinder the complexation of calcium ions with sodium alginate, due to the ability of the latter to interact with whey proteins via electrostatic attraction forces leading to the formation of self-assembled particle aggregates (Fioramonti, Perez, Aríngoli, Rubiolo, & Santiago, 2014). The formed alginate-whey protein aggregates do not provide sufficient protection against the harsh gastric juice conditions as they are not able to encapsulate the bacterial cells explaining the significantly lower (*p* < 0.01) viabilities of *L. rhamnosus* GG in the case of WPC based films. Our results indicate one medium size (30–40 g) slice of bread can deliver approx. 7.57–8.98 and 6.55–6.91 log cfu/portion before and after the in-vitro digestion respectively, meeting the required minimum amount of viable probiotic bacteria delivered to the human host (Saad et al., 2013).

### 3.5 Determination of bread staling by DSC

To evaluate the possible effects of the edible films on bread staling, bread crust and crumb samples were subjected to DSC analysis (Tables 2 and 3). It is well established that the ice fusion enthalpy of bread crumb is associated with water available for starch retrogradation (Ribotta & LeBail, 2007). According to DSC analysis of the bread crumb samples (Table 2), the application of edible films is accompanied by a significant (*p* < 0.001) reduction of the ice fusion enthalpy and freezing point temperature of the samples compared to the control ones. The increase of drying temperature induced a further reduction of ice fusion enthalpy due to the extended water removal. The presence of whey proteins promoted the decrease of the unbound water possibly due to the hygroscopic properties of lactose (Soukoulis et al., 2013). In order to evaluate the impact of unfreezable water on the staling of bread loaves, bread crust samples were analysed after 7 days of storage (Table 3). According to the obtained thermograms, a significant phase transition at −30.9 to −31.9 °C was observed which can be attributed to the glass transition of the crust systems. Moreover, two endothermic peaks, first at 48.4–51.1 °C and second at 154–161 °C were also detected and attributed to starch retrogradation and the formation lipid–amylose complexes (Czuchajowska & Pomeranz, 1989). No significant effect on the amount of starch retrogradation and amylose–lipid enthalpies was observed in terms of film type or storage temperature. Thus, it can be proposed that the presence of the film would have a rather limited impact on bread staling after 7 days.

### 3.6 Textural characteristics of the bread crust

Bread staling is well known as being associated with major changes in the textural qualities of both bread crust and crumb. Despite its complexity, crumb staling has been widely studied due to its direct relationship with physicochemical and colloidal changes that occur during storage, such as water migration or amylopectin retrogradation (Gray & Bemiller, 2003).

**Fig. 8:**
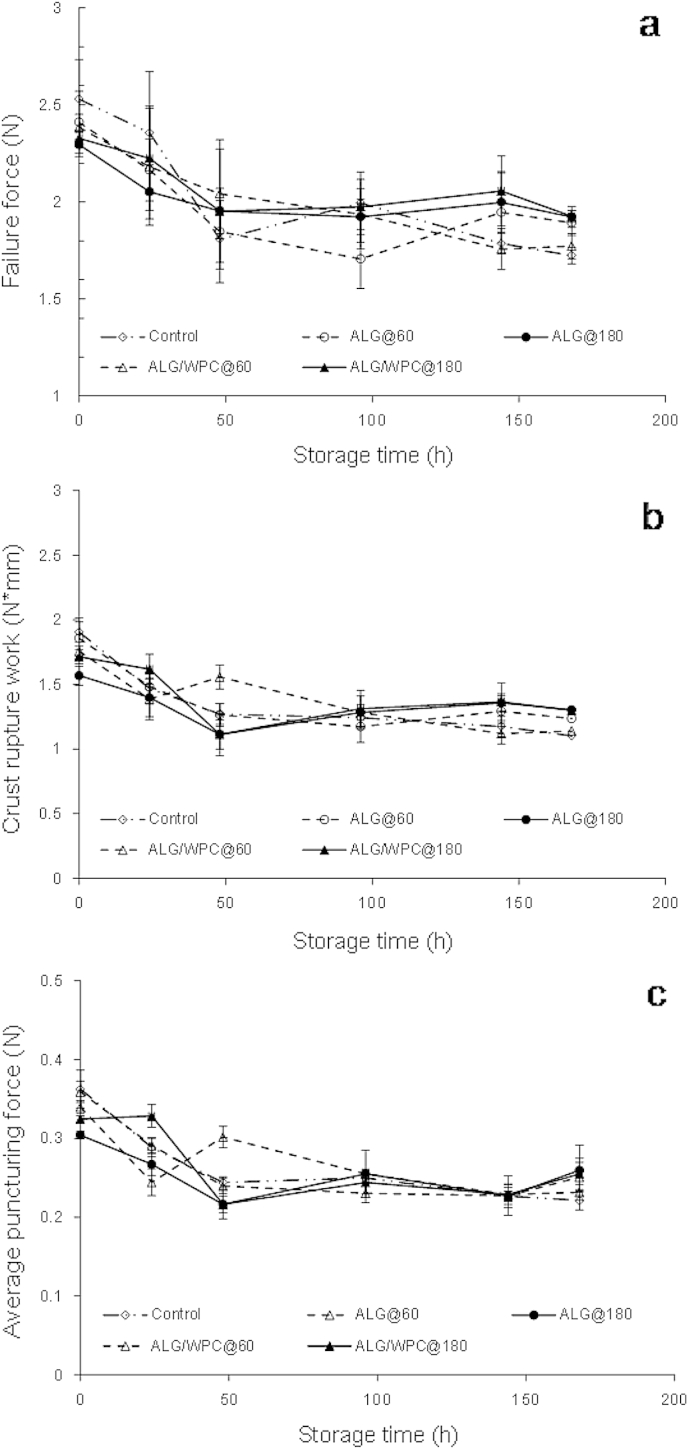
Textural characteristics changes of probiotic pan bread crust coated with sodium alginate (ALG) or sodium alginate–whey protein concentrate (ALG/WPC) edible films and air dried either at 60 °C for 10 min or 180 °C for 2 min, during 7 days (168 h) of storage at room temperature.

There was no significant impact (*p* > 0.05) of the film composition or the film drying method (Fig. 8) on textural parameters and storage time influenced significantly (*p* < 0.01) all the textural properties associated with the puncture tests. A steep decrease of the failure and average puncturing force as well as of the crust rupture work values was observed which indicates loss of crispness and the formation of a soft leathery bread crust (Gray & Bemiller, 2003).

### 3.7 Flavour profiling of the probiotic bread crust samples

In order to monitor the possible changes of the crust flavour profile of the bread crust due to the presence of the probiotic edible films, APCI-MS headspace analysis of the bread crust samples 2 h and 7 days after the baking process was conducted. According to the acquired APCI-MS mass fingerprints for the plain and probiotic bread crusts, more than 25 volatile compounds were tentatively identified based on their fragmentation pattern as well as using historical literature data relating to bread crust flavour profiles (Table 4). Fifteen flavour compounds were also detected and identified using SPME-GC-MS headspace analysis of freshly made control and probiotic bread crust samples, this is reported in Table 4.

Regardless of bread type and storage time, protonated nominal mass ions (*m*/*z*) 43, 47, 59, 61, 75, 81, 83, 87, 89, 93, 97, 101, 113, 121, and 143 had the highest intensities. This is generally in agreement with the findings of Heenan, Dufour, Hamid, Harvey, and Delahunty (2009) who fingerprinted the flavour profile of different bakery products using proton transfer reaction mass spectrometry.

**Fig. 9:**
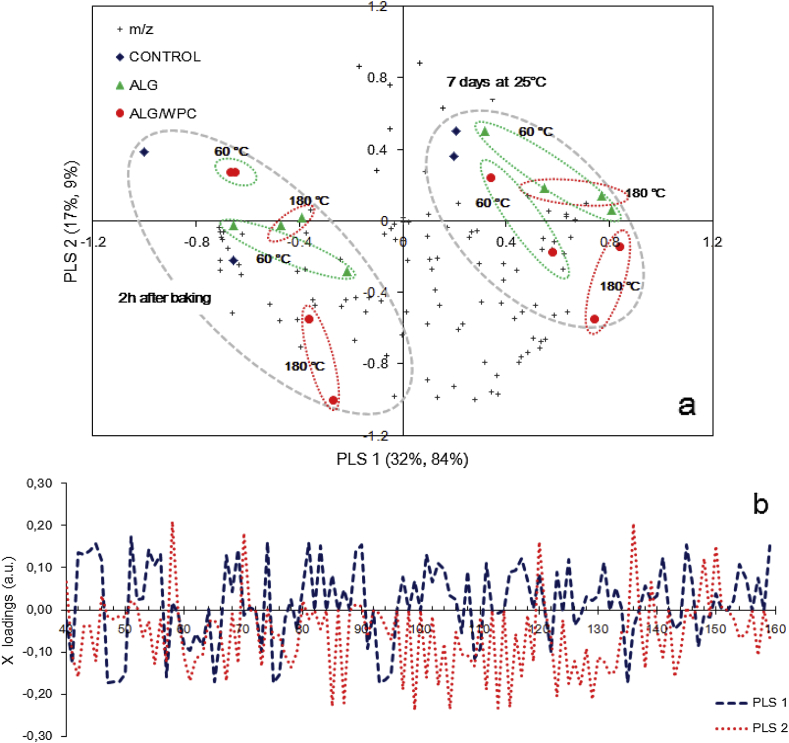
Partial least squares regression analysis of the mass spectral fingerprint (*m*/*z* 40–160) acquired during APCI-MS headspace analysis of probiotic pan bread crust samples 2 h after baking and 7 days of storage at room temperature. a: classification of samples based on edible film characteristics and storage time and b: PLS loadings plot for the two first principal components axes (ALG = sodium alginate, WPC = whey protein concentrate).

In order to identify possible APCI-MS markers associated with the flavour profile changes throughout the bread storage, the entire spectral dataset (*m*/*z* 40–160) were subjected to PLS analysis (Fig. 9). As can be seen, the storage time of both plain and probiotic bread systems had a prominent impact on their flavour profile. Apart from the bread crust samples coated with the WPC based films and dried at 180 °C, the presence of probiotic edible films did not influence bread crust flavour. Bread crust samples were fully discriminated using the first three PLS axes, whereas based on the X-loadings plot (Fig. 9b), twenty protonated mass ions were significantly (*p* < 0.05) associated with flavour changes due to bread staling. According to sample clustering (Fig. 9a), PLS-1 axis was mainly related with changes of the volatile compounds present in the headspace above bread crust during ageing, whilst PLS-2 discriminates the WPC coated breads dried at 180 °C from the plain and the rest probiotic bread systems. Bread staling was accompanied by a significant increase of the intensity of *m*/*z* 43, 45, 71, 73, 83, 85, 89, 97, 101, 141, 143 and 145. Moreover, a reduction of the signal for *m*/*z* 41, 47, 49, 57, 61, 65, 75, 95, 121, and 135 was also observed after 7 days of storage at room temperature. The changes in the volatile compounds composition of bread crust are primarily associated with their initial concentration, their partition coefficients and volatility, the interactions with other components and the bread matrix (Jensen, Oestdal, Skibsted, Larsen, & Thybo, 2011; Pozo-Bayón, Guichard, & Cayot, 2006). To identity the discriminating mass ions in terms of storage time (Table 4), *m*/*z* 47, 61, 75, 83, 85, 89, 101, 107, 143, 145 could be attributed to protonated molecules of ethanol, acetic acid, propionic acid, 1-hexanol, hexanal, diacetyl, acetoin, hexanal/2,3-pentanedione, benzaldehyde, nonanal and ethyl hexanoate respectively. Moreover, *m*/*z* 45, 59, and 73 are widely accepted as being the protonated mass ions of acetaldehyde, acetone and 2-butanone/butanal (Gkatzionis, Linforth, & Dodd, 2009; Yu et al., 2012). PLS analysis results suggest that when compared oven the storage time both conventional and probiotic bread crust samples experienced a depletion of their major flavour compounds associated with dough fermentation e.g. ethanol, acetic and propionic acid whilst a pronounced increase of the compounds related mainly with carbonyl compounds (acetaldehyde, butanal/2-butanone, hexanal/2-hexanone, nonanal), alcohols (2-, 3-methyl-butanol, 1-hexanol), acetoin, and ethyl hexanoate was observed. The latter compounds could be either attributed to the metabolic activity of the *L. rhamnosus* GG e.g. acetaldehyde, acetoin and 2,3-pentanedione formation or to lipid oxidation products e.g. butanal, pentanal, hexanal and nonanal (Jensen et al., 2011; Soukoulis et al., 2010). Finally, some volatile compounds e.g. diacetyl (*m*/*z* 79), acetone (*m*/*z* 59) although they were detected at significantly high amounts in the headspace of bread crust samples did not exhibit any remarkable change as function of storage time.

Regarding the impact of the edible film type and drying method, only bread crust samples coated with the WPC – sodium alginate film forming solutions and dried at oven temperature exhibited a pronouncedly different flavour profile (Fig. 9b), which could be attributed to the formation of Maillard reaction volatile products. However, it should be noted that the complexity of the acquired fingerprints did not allow us to identify any potential marker of Maillard reactions.

## 4 Conclusions

The development of probiotic breads through the use of air dried probiotic edible films was found to be successful. Correct design of the film forming solution has been shown to be a critical factor for the viability of *L. rhamnosus* GG during the air drying step, room temperature storage and simulated in-vitro digestion. The presence of whey proteins in the film forming solution reduced *L. rhamnosus* GG viability losses throughout drying and storage and the use of film systems based exclusively on sodium alginate exhibited a very good performance under in-vitro digestion, this is proposed to be due to an ionic setting mechanism. The former was attributed to the ability of *L. rhamnosus* GG to interact with whey proteins and therefore reducing the osmotic, heat or oxidative stresses that occur in the system. The presence of edible films did not modify the textural, flavour and thermophysical properties of bread crust samples indicating similar mechanisms of bread staling took place in both conventional and probiotic breads.

## Figures and Tables

**Table 1:** Colour characteristics of conventional and probiotic bread crust samples coated with sodium alginate (ALG) or sodium alginate–whey protein concentrate (ALG/WPC) edible films and air dried either at 60 °C for 10 min or 180 °C for 2 min.

	L*	a*	b*	ΔΕ*
Control	68.9 ± 0.2c	10.3 ± 0.6a	31.1 ± 0.2b	
ALG@60 °C	68.4 ± 0.3c	11.7 ± 0.3ab	30.8 ± 0.9ab	2.47 ± 0.2a
ALG@180 °C	67.4 ± 0.3b	11.8 ± 0.3ab	32.2 ± 1.0b	2.98 ± 0.7a
ALG/WPC@60 °C	66.9 ± 0.6b	11.4 ± 0.4ab	31.2 ± 0.5b	2.80 ± 0.1a
ALG/WPC@180 °C	64.0 ± 0.2a	13.1 ± 0.2b	29.5 ± 1.1a	6.55 ± 0.4b

a–c Different letter between rows indicates significantly different values (*p* < 0.05) according to Duncan's post hoc means comparison test.

**Table 2:** Thermophysical properties of the adjacent to crust bread crumb samples 2 h after baking (ALG = sodium alginate, WPC = whey protein concentrate).

	*T*_*m*,onset_	*T*_*m*_	Δ*H*_*m*_
Control	−11.5 ± 0.3a	−2.6 ± 0.2a	53.8 ± 4.9d
ALG@60 °C	−12.3 ± 0.5a	−3.7 ± 0.7b	39.5 ± 2.1c
ALG@180 °C	−14.3 ± 0.6b	−5.4 ± 0.5c	29.4 ± 1.1b
ALG/WPC@60 °C	−15.0 ± 0.1c	−6.1 ± 0.0d	28.1 ± 2.4b
ALG/WPC@180 °C	−15.5 ± 0.1d	−6.4 ± 0.0e	20.1 ± 0.6a

a–d Different letter between rows indicates significantly different values (*p* < 0.05) according to Duncan's post hoc means comparison test.

**Table 3:** Thermophysical properties of bread crust samples after 7 days of storage at 25 °C (ALG = sodium alginate, WPC = whey protein concentrate).

	*T*_*g*,onset_	*T*_*g*,mid_	Δ*C*_*p*_
Control	−34.8 ± 0.3a	−31.9 ± 0.1a	0.112 ± 0.004a
ALG@60 °C	−33.9 ± 0.1a	−30.9 ± 0.3a	0.107 ± 0.002a
ALG@180 °C	−34.1 ± 0.0a	−31.4 ± 0.1a	0.106 ± 0.001a
ALG/WPC@60 °C	−34.8 ± 0.3a	−31.9 ± 0.1a	0.164 ± 0.018b
ALG/WPC@180 °C	−34.7 ± 0.2a	−31.7 ± 0.3a	0.119 ± 0.004a

	*T*_retro,onset_	*T*_retro,mid_	Δ*H*_retrogradation_
Control	33.9 ± 0.1b	49.1 ± 0.0ab	1.43 ± 0.17a
ALG@60 °C	36.3 ± 1.1b	49.7 ± 0.2ab	1.74 ± 0.11a
ALG@180 °C	38.3 ± 1.5b	51.1 ± 0.5c	1.55 ± 0.02a
ALG/WPC@60 °C	34.5 ± 0.3b	50.4 ± 0.1bc	1.22 ± 0.13a
ALG/WPC@180 °C	29.4 ± 1.1a	48.4 ± 0.2a	1.74 ± 0.11a

	*T*_amyl–lip,onset_	*T*_amyl–lip,mid_	Δ*H*_amylose–lipid_
Control	139 ± 1.8a	155 ± 0.1ab	3.97 ± 0.69a
ALG@60 °C	137 ± 2.6a	154 ± 0.0a	5.65 ± 0.26a
ALG@180 °C	141 ± 0.7a	158 ± 1.6bc	3.87 ± 0.67a
ALG/WPC@60 °C	142 ± 0.4a	161 ± 0.0c	4.43 ± 0.24a
ALG/WPC@180 °C	139 ± 0.2a	155 ± 0.1ab	3.55 ± 0.16a

a–c Different letter between rows indicates significantly different values (*p* < 0.05) according to Duncan's post hoc means comparison test.

**Table 4:** Identification of the protonated molecular ions.

Protonated molecular ion (*m* + 1)	Compound identification	Odour description^a^	Identification method	Reference
41	Alcohol (DF)^b^	n/a	Tentative	n/a
43	Alcohol (DF)	n/a	Tentative	n/a
45	Acetaldehyde	Pungent, ethereal odour	Tentative	
47	Ethanol	Alcohol	SPME-GC-MS	[1–4]
57	1-Butanol	Solvent, fusel-like, burning taste	Tentative	[1,2]
59	2-Propanone	Pungent, solvent, sweet	Tentative	[1–4]
61	Acetic acid	Acid, pungent	SPME-GC-MS	[1,2]
71	3-methyl-butanol, (DF)	Malt	SPME-GC-MS	[1,2]
	2-methyl-butanol	Malt	SPME-GC-MS	[1,2]
	3-hydroxy-2-butanone	Yogurt, fatty, butter	SPME-GC-MS	[1–4]
73	2-Butanone	Sweet apricot	Tentative	[1,2]
75	Propionic acid	Pungent, rancid	Tentative	[1,2]
81	Furfuryl alcohol	Burnt	Tentative	[1,2]
83	Hexanal (DF)	Green, tallow	SPME-GC-MS	[1–2,4]
85	1-Hexanol (DF)	Herbaceous, sweet, green fruit	SPME-GC-MS	[1–2,4]
87	2,3-Butanedione	Butter	SPME-GC-MS	[1–4]
89	3-hydroxy-2-butanone	Yogurt, fatty, butter	SPME-GC-MS	[1–4]
95	Dimethyldisulfide	Onion, garlic, cheese, savoury flavour	Tentative	[2]
97	2-Furfural	Almond, bread	Tentative	[1–2,4]
101	Hexanal	Green, tallow	SPME-GC-MS	[1,2]
	2,3-pentanedione	Sweet, quinone like, buttery	Tentative	[2]
107	Benzaldehyde	Almond, caramel	SPME-GC-MS	[1,2]
143	Nonanal	Citrus, fat, soap	SPME-GC-MS	[1,2]
145	Ethyl hexanoate	Fruit, apple peel	Tentative	[1,2]

[1] = Jensen et al. (2011), [2] = Bianchi, Careri, Chiavaro, Musci, and Vittadini (2008), [3] = Heenan et al. (2009), [4] = Plessas et al. (2008).

a Burdock (2002).

b DF = dehydrated molecular ion fragment.
